# Reasons for Hospitalization of Psoriasis Patients: A Report From the National Inpatient Sample

**DOI:** 10.7759/cureus.12271

**Published:** 2020-12-25

**Authors:** Karun M Nair, Sandhya Shri Kannayiram, Armaan Guraya, Osahon N Idolor, Osaigbokan P Aihie, Eseosa J Sanwo, Chukwudi C Muojieje

**Affiliations:** 1 Internal Medicine, John H. Stroger, Jr. Hospital of Cook County, Chicago, USA; 2 Medicine, Midwestern University Chicago College of Osteopathic Medicine, Downers Grove, USA; 3 College of Medicine, University of Benin, Benin, NGA; 4 School of Medicine, University of Missouri, Columbia, USA; 5 Internal Medicine, MountainView Regional Medical Center, Las Cruces, USA

**Keywords:** psoriasis, hospitalization, sepsis, infection, cardiovascular, mortality, national inpatient sample

## Abstract

Background

We used a large United States population-based database to analyze the reasons for hospitalization of psoriasis patients.

Methods

International Classification of Diseases, 10th revision (ICD-10) code was used to identify hospitalizations in National Inpatient Sample (NIS) 2017 with a principal or secondary diagnosis of psoriasis. The reasons for hospitalization were divided into 19 categories based on their principal discharge ICD-10 diagnosis code. We also ranked the five most common specific reasons for hospitalization of psoriasis patients.

Results

There were over 35 million discharges included in the 2017 NIS database. A total of 165215 hospitalizations had either a principal or secondary ICD 10 code for psoriasis. Based on ICD-10 code categories, the top five reasons for hospitalization in patients with history of psoriasis were: Cardiovascular (CV) (26605, 16.10%), rheumatologic (19555, 11.84%), digestive (18465, 11.18%), infection (16395, 9.92%), and respiratory (14865, 9.00%). Sepsis was the most common principal diagnosis of psoriasis hospitalizations.

Conclusion

CV diseases were the most common ICD category, and sepsis was the most common principal diagnosis for psoriasis hospitalization. Management of medical co-morbidities is important in reducing rates of hospitalization of psoriasis patients.

## Introduction

Psoriasis is a chronic inflammatory autoimmune disorder of the skin affecting over 2% of the US population. It increases incidence with age and frequency among Caucasians compared to other ethnic groups and no clear inclination towards gender [1,2]. Although initially, psoriasis was thought to be a disease of the skin, it has become increasingly recognized as a systemic inflammatory disease resulting in multiple comorbidities, especially with longer duration and severity of the disease [3,4]. Psoriasis has been found to significantly reduce life quality, affecting physical and mental health with an increased risk of poor self-esteem, anxiety, depression, and substance use [5].

Over the last decade, abundant research has been done on psoriasis and its comorbidities, particularly elucidating the association and increased risk of cardiovascular (CV) disease and major adverse cardiac events (MACE) with psoriasis [6,7]. Severe psoriasis, by itself, was found to be an independent risk factor of cardiovascular disease [8]. Apart from cardiovascular diseases, psoriasis is also associated with malignancy, serious infections, other autoimmune disorders, nonalcoholic fatty liver disease, chronic obstructive pulmonary disease, and chronic kidney disease [4,9,10]. The association of psoriasis with multiple comorbidities is thought to be due to shared genetics, pathophysiology of systemic inflammation, and the increased prevalence of risk factors like smoking, obesity, and alcohol use in psoriasis patients [11]. Psoriasis is a systemic disease associated with multiple medical co-morbidities such as atrial fibrillation and other CV diseases, which increases the risk of hospitalization [12].

A few studies have been done to show the all-cause and cause-specific mortality in psoriasis, including population-based studies in Sweden demonstrating the increased risk of death due to its multiple comorbidities [13]. But limited national-level data is available in the United States (US) on reasons for hospitalizations of psoriasis patients. Hence, in this report, we used the National Inpatient Sample, a large United States population-based database, to answer this clinically relevant question.

## Materials and methods

Data source

Using the International Classification of Diseases, 10th revision (ICD-10) code, we searched for hospitalizations with psoriasis as the principal or secondary diagnosis in National Inpatient Sample (NIS) 2017. NIS is the largest inpatient admission database in the United States [14-17]. NIS is a 20% probability sampling of different strata, structured to be representative at the national level [18-21]. Each hospitalization in the 2017 NIS has a principal diagnosis and up to 40 secondary diagnoses [22]. The principal diagnosis is the main reason for hospitalization, while the remaining diagnoses are secondary diagnoses [23]. Since patient data in NIS is de-identified, and the NIS database is available to the public, this study was exempted from institutional board review.

Inclusion and exclusion criteria

The study population consisted of all hospitalizations in 2017 with a principal or secondary psoriasis ICD-10 diagnosis code. We excluded hospitalizations for patients less than 18 years. We used ICD-10 code “L04” to identify hospitalizations with a principal or secondary diagnosis code for psoriasis.

Statistical analysis

Analyses were performed using statistical and data (STATA, version 16, StataCorp LLC, College Station, Texas, USA).

Outcomes

The total number of psoriasis discharges, age, race, hospital length of stay (LOS), and total hospital charges were recorded. The reasons for hospitalizations were divided into 19 categories based on their principal discharge ICD-10 diagnosis code. We also ranked the five most common specific reasons for hospitalizations. The principal diagnosis of hospitalization was taken to be the reason for hospitalization.

## Results

There were over 35 million discharges included in the 2017 NIS database. A total of 165215 hospitalizations had either a principal or secondary ICD-10 code for psoriasis. Patients were predominantly white, had equal sex distribution, average age of 60.8 years, LOS was 5.5 days and average total hospital charge was $58,272. The top five reason for psoriasis hospitalization by ICD-10 code categories in descending order of frequency were as follows (Table 1): Cardiovascular (26605, 16.10%), rheumatologic (19555, 11.84%), digestive (18465, 11.18%), infection (16395, 9.92%), respiratory (14865, 9.00%). Sepsis was the most common principal diagnosis of psoriasis hospitalizations, followed by primary knee osteoarthritis, acute chronic obstructive pulmonary disease (COPD) exacerbation, pneumonia, non-ST elevation myocardial infarction, and acute kidney injury in descending order of frequency. Cellulitis was the only skin disease and subcutaneous tissue disorder among the top 25 reasons for hospitalization.

**Table 1:** ICD-10 code categories of adult psoriasis hospitalizations.

ICD-10 Code Admission Category	Admission Category	Number of Admissions
Certain infections and parasitic diseases A00-B99	Infections	16395
Neoplasms & diseases of the blood and blood-forming organs and certain disorders involving the immune mechanism C00-D49 & D50-D89	Hema/Onc	8010
Endocrine, nutritional and metabolic diseases E00-E89	Endo	7115
Mental, behavioral and neurodevelopmental disorders F01-F99	Psychiatry	9675
Diseases of the nervous system G00-G99	Neuro	4040
Diseases of the eye and adnexa and ear H60-H95 H00-H59	Eye/Ear	430
Diseases of the circulatory system I00-I99	CVS	26605
Diseases of the respiratory system J00-J99	Resp	14865
Diseases of the digestive system K00-K95	GI	18465
Diseases of the skin and subcutaneous tissue L00-L99	Skin	9520
Diseases of the musculoskeletal system and connective tissue M00-M99	Rheum	19555
Diseases of the genitourinary system N00-N99	GU	7070
Pregnancy, childbirth, and puerperium O00- O9A	OB	2735
Certain conditions originating in the perinatal period P00-P96	Perinatal	0
Congenital malformations, deformations, and chromosomal abnormalities Q00-Q99	Congenital	255
Symptoms, signs, and abnormal clinical laboratory findings, not elsewhere classified R00-R99	Other	4885
Injury, poisoning, and certain other consequences of external causes S00-T88	Injury/Poison	13710
External causes of morbidity (accidents & violence) V00-Y99	Accidents	0
Factors influencing health status and contact with health services Z00-Z99	Health Services	1880

## Discussion

To better characterize the reasons for hospitalization in patients with psoriasis, we conducted a review of the NIS database. This information will assist us in practicing sufficient preventative care to optimize outcomes in these patients. We found that the greatest proportion of hospitalizations was attributed to cardiovascular (CV) causes, followed by rheumatologic, digestive, infectious, and respiratory categories.

There exists an established link between psoriasis and cardiovascular morbidity and mortality, which is reflected in the relative high proportion of CV admissions in psoriasis patients, at 16.1%. Underlying mechanisms of systemic immune deregulation in psoriasis can contribute to cardiovascular injury. Gaeta et al. reported a 25% increased relative risk of CV disease in patients with psoriasis independent of smoking, obesity, and hyperlipidemia [24]. A study by Gelfand et al. provided strong evidence suggesting that psoriasis is an independent risk factor for myocardial infarction [6]. This United Kingdom (UK) prospective study showed the adjusted relative risk for myocardial infarction was 1.29 (95% confidence interval CI, 1.14-1.46) for a 30-year-old patient with mild psoriasis and 3.10 (95% CI, 1.98-4.86) if psoriasis was severe compared to similar patients without psoriasis. Further studies have elucidated possible mechanisms for this link, including Flammer and Ruschitzka who demonstrated similar histological features between the dermatologic lesions associated with psoriasis and vascular plaques characteristic of atherosclerosis, suggesting a common pathogenesis [25]. CV diseases were found to be the most common ICD-10 category for admission in our study. This reinforces the correlation between psoriasis and CV-related hospitalization and morbidity.

Rheumatologic causes accounted for the second highest frequency for hospitalization by ICD-10 code category accounting for 11.84% of admissions overall. A UK-based electronic medical record study [26] showed that psoriasis overall was associated with higher prevalence of autoimmune rheumatologic diseases beyond psoriatic arthritis (OR: 2.04; 1.71-2.42, p < 0.001). Trend analysis revealed significant associations between psoriasis severity and rheumatologic diseases (p < 0.05).

Primary knee osteoarthritis, more specifically, was found to be the second most common principal diagnosis. Joint disease is often implicated in patients with psoriasis, with up to 23.8% prevalence of psoriatic arthritis in psoriasis patients per meta-analysis conducted by Alinaghi et al. [27]. However, non-inflammatory joint disease such as primary knee osteoarthritis appears to have an especially elevated burden in terms of hospitalization for patients with psoriasis according to the NIS database.

While infectious diseases represented 9.92% of admissions, ranking fourth in terms of categories for hospitalization, sepsis was the most common primary diagnosis for hospitalization. This susceptibility to infection may possibly be explained by use of certain biologic agents, as the PSOLAR study demonstrated a higher risk of serious infections in patients being treated with adalimumab and infliximab compared to nonbiologic therapies [28]. These data represent important factors that may inform decision-making strategies for immunosuppressive therapy for patients with psoriasis, and guide further studies comparing therapy approach and risk of serious infection.

Psoriasis carries an increased risk of all-cause mortality according to a meta-analysis conducted by Dhana et al., as well as cause-specific mortality for cardiovascular, liver, renal, neoplastic, and lower respiratory tract diseases, which had increasing risk relative to disease severity [29]. Mechanisms underlying this increased risk of mortality have yet to be fully elucidated. It is thereby constructive to identify and manage comorbidities early in patients with psoriasis in order to prevent hospitalization and mortality.

The large sample size and nature of the NIS which enables us to categorize reasons for hospitalizations into different ICD-10 organ-system based categories and specific reasons is the major strength of the study.

There are some limitations to our study: i) the NIS is an administrative database that uses claims data via ICD-10 codes to characterize diagnoses, hence there is a possibility of errors associated with coding. ii) This report reflects data on hospitalizations rather than on individual patients.

## Conclusions

In patients with a history of psoriasis, CV diseases were the most common ICD-10 code category for hospitalization, while sepsis was the most common principal diagnosis for hospitalization. Adequate management of medical comorbidities (e.g., CV) is needed to reduce the rate of hospitalization.
